# Inhibition of liver fibrosis using vitamin A-coupled liposomes to deliver matrix metalloproteinase-2 siRNA *in vitro*

**DOI:** 10.3892/mmr.2015.3842

**Published:** 2015-05-25

**Authors:** YIPING LI, FENG LIU, FENGAN DING, PINGSHENG CHEN, MENG TANG

**Affiliations:** 1 Department of Pathology, Medical School of Southeast University, Jiangsu 210009, P.R. China; 2 Department of Toxicology, School of Public Health, Southeast University, Nanjing, Jiangsu 210009, P.R. China

**Keywords:** cationic liposomes, hepatic stellate cells, liver fibrosis, matrix metalloproteinase-2, small interference RNA

## Abstract

Hepatic fibrosis is a common form of wound healing in response to chronic liver injuries and can lead to more serious complications, including mortality. It is well-established that hepatic stellate cells (HSCs) are central mediators of hepatic fibrosis, and matrix metalloproteinase-2 (MMP-2) is important in the formation of liver fibrosis. In addition, HSCs are the primary cells secreting MMP-2 and extracellular matrix, therefore, there has been increasing interest in developing agents with high selectivity towards HSCs. However, no clinical drugs based on MMP-2, directed against HSCs, have been used to prevent fibrosis. Following consideration of the abundant vitamin A (VitA) receptors expressed on the cellular membrane of HSCs, the present study constructed VitA-coupled liposomes (VitA-lips) using dicyclohexylcarbodiimide-1, 3-diaminopentane condensation, rotatory film processing and ultrasonic oscillation. The results revealed that the liposomes exhibited low cytotoxicity and a suitable binding ability to MMP-2 small interference (si)RNA. Furthermore, the liposomes effectively delivered MMP-2 siRNA to the HSC-T6 cells. When HSCs were treated with the liposomes carrying MMP-2 siRNA (VitA-lip-MMP-2 siRNA), the mRNA expression and activity of MMP-2, and the protein expression levels of α-smooth muscle actin and type I collagen were significantly reduced. These results suggested that inhibition of the expression of MMP-2 in HSC-T6 cells may contribute to preventing hepatic fibrosis, and provided experimental support to the development of specific drugs against MMP-2 to prevent fibrogenesis in chronic liver disease.

## Introduction

Hepatic fibrosis, a prominent pathological feature at the end stage of chronic liver disease, is a dynamic process and develops due to an increase in extracellular matrix (ECM) synthesis and deposition, along with insufficient remodeling, however, fibrosis may be reversible prior to the establishment of advanced architecture (1–3). Matrix metalloproteinase-2 (MMP-2) may be involved in the formation and reversal of hepatic fibrosis (4–6). MMP-2 promotes the development of fibrosis in the early stage, but increased MMP-2 activity can induce the reversal of fibrosis following removal of the pathogenic factors (4,6). However, whether MMP-2 has an effect on the formation of hepatic fibrosis by directly examining hepatic stellate cells (HSCs) remains to be elucidated, and no clinical antifibrotic drugs based on MMP-2 have been approved for preventing fibrosis (5,7).

RNA interference (RNAi) is a strategy, which involves specifically degrading homogenous mRNA to suppress its gene expression. Compared with other techniques for inhibiting gene expression, this method is not only specific, effective and persistent, but is also simple and safe. However, the vectors for siRNA have certain bottlenecks, including low transfer efficiency and inevitable prominent toxicity. Among these vectors, cationic liposomes are notable due to their lack of immunogenicity and natural degradation, however, specific targeting remains a challenge (8).

Several studies have demonstrated that hepatic stellate cells (HSCs) are crucial mediators of fibrosis (3,9,10). HSCs reside in the perisinusoidal space and store vitamin A (VitA) in their quiescent state (11). The present study selected HSCs as target cells of fibrosis to analyze the effect of VitA-coupled liposomes (VitA-lips) combined with MMP-2 siRNA, which may aid further investigations examining specific fibrosis prophylactic drug treatments.

## Materials and methods

### Materials

Cholesterol (Aobox, Peiking, China), 3β-[N -(N′,N′-dimethylaminoethane) carbamoyl] cholesterol (DC-Chol; Sigma-Aldrich, St. Louis, MO, USA), 1,2-Dioleoyl-sn-glycero-3-phosphoethanolamine (DOPE; Sigma-Aldrich), MMP-2-siRNA (GenePharma, Shanghai, China), 4′,6-diamidino-2-phenylindole (DAPI) staining solution (Beyotime Institute of Biotechnology, Haimen, China), mouse anti-human α-SMA monoclonal antibody (cat. no. ZM-0003), rabbit anti-mouse type I collagen monoclonal antibody (cat. no. ZA-0616) and immunohistological reagent kits (Zhongshan Golden Bridge Biotechnology Co., Ltd., Beijing, China), gelatin zymography assay kits (Applygen Technologies, Inc., Beijing, China), H2600 transmission electronic microscope (TEM; JEM-200CX; JEOL, Ltd., Tokyo, Japan), Zeta Sizer 3000 laser particle size analyzer (Malvern Instruments Ltd., Malvern, UK), high-pressure homogenizer (Avestin, Ottawa, Canada), and fluorescent microscope (TI-S; Nikon Corporation, Tokyo, Japan) and an inverted fluorescent microscope (TE2000-U, Nikon Corporation). were all used. The hepatic stellate cell line HSC-T6 was purchased from the Cell Bank of the Chinese Academy of Sciences (Shanghai, China).

### Cell culture

The HSC-T6 rat hepatic stellate cells were grown in Dulbecco's modified Eagle's medium (DMEM, Gibco Life Technologies, Carlsbad, CA, USA), supplemented with 10% (v/v) fetal bovine serum (Gibco Life Technologies) and antibiotics (100 U/ml streptomycin and 100 U/ml penicillin) in plastic culture flasks or dishes at 37°C in a humidified incubator under a 5% CO_2_ atmosphere, and the culture medium was replaced with fresh medium every other day. The confluent cells were then harvested with 0.25% trypsin-EDTA solution.

### Preparation of cationic liposomes

DC-Chol, DOPE and cholesterol (4:3:3, molar ratio) were mixed in round-bottom flasks, and CCl_3_-CH_3_OH (3:2, v/v) was added to the mixture, followed by sonication for 5 min in a water bath prior to evaporation in 37°C to remove organic reagents. Finally, homogenous thin films were obtained from the internal walls of the flasks. The films were resolved with 20 ml phosphate-buffered saline (PBS; pH 7.2), followed by centrifugation for 30 min at room temperature (RT). When the films were completely stripped down, they were sonicated for 1 min and then squeezed three times through 220 nm filters (Merck Millipore, Darmstadt, Germany) to reduce their size. Simultaneously, VitA-cephalin was synthesized using the dicyclohexylcarbodiimide-1, 3-diaminopentane (DCC-DAMP) method. Briefly, 50 mg VitA (Zhengzhou Lion Biological Technology Co., Ltd., Henan, China) and 100 mg phycoerythrin (Sigma-Aldrich) were dissolved in 5 ml dimethyl sulfoxide (DMSO; Sigma-Aldrich), and mixed with 100 *μ*l DAMP (Sinopharm Chemical Reagent Beijing Co., Ltd., Beijing, China), followed by the activation at 4°C for 30 min. Subsequently, DCC (Sinopharm Chemical Reagent Beijing Co., Ltd.) solution (50 mg in 1 ml chloroform) was slowly added to the above mixture, and agitated for 24 h at RT in the dark. After standing for 2 h at RT, the supernatant were mixed with 20 ml cold acetone (Sinopharm Chemical Reagent Beijing Co., Ltd.), and centrifuged for 10 min at 9,279 x g at RT. The precipitate was washed with cold acetone for three times and dried at RT, then the products were analyzed using a Fourier Transform Infrared spectrometer (Avatar 370 Thermo Nicolet; Thermo-Nicolet Corporation, Madison, WI, USA).

### Characterization of cationic liposomes

Cationic liposomes were diluted and dropped onto copper gauze with a membrane for morphological characterization. The particle size was measured using a Zeta Sizer 3000 laser particle size analyzer and processed using Dynamic Light Scattering software (Malvern Instruments Ltd.). The average particle size and polydispersity index (PDI) were recorded. No aggregation or deposition were observed in the cationic liposomes on being maintained at RT for 2 months, and the stability was further examined using TEM. Briefly, a few drops of the liposome solution were dropped onto a TEM grid, dried and absorbed the extra solution with filter paper prior to analysis. TEM images were photographed using a field emission JEM-200CX TEM equipped with a charge-coupled device camera.

### Reverse transcription-quantitative polymerase chain reaction (RT-qPCR)

The VitA-lip-MMP-2 siRNA complex (mass ratio of cationic liposome to MMP-2-siRNA, 5:1; siRNA, 30 nM) was used to transfect the HSC-T6 cells at a density of 70–80%. Subsequent to the cells being transfected for 36 h at 37°C, the total RNA was extracted using TRIzol^®^ reagent (Invitrogen Life Technologies, Carlsbad, CA, USA), and the mRNA expression of MMP-2 was analyzed using RT-qPCR. Briefly, 1 *μ*g total RNA was reverse transcribed in a reaction mix containing 2 ml dithiothreitol (0.1 M), 1 *μ*l dNTPs (100 mM), 2 *μ*g random hexamers, 1 *μ*l (200 units) superscript II reverse transcriptase and 1 *μ*l (40 units) RNAse inhibitor (GE Healthcare, Chalfont, UK) for 1 h at RT, then the synthesized cDNA was used for RT-qPCR. The sequences of the sense and antisense primers for MMP-2 were 5′-CATCGTACTCCTCGTTGCTGATCCACAT-3′ and 5′-CTCCCTCATGCCATCCTGCGTCTG-3′, respectively. To normalize the loading samples, a GAPDH control was produced using sense 5′-GAAGGGCTCATGACCACAGT-3′ and antisense 5′-GGATGCAGGGATGATGTTCT-3′ primers. The primers were synthesized by Yingjun Biotechnogy Co., Ltd. (Shanghai, China). The PCR amplification cycles were as follows: 94°C 5 min, followed by 35 cycles of 94°C for 30 sec, 62.5°C for 30 sec and 72°C for 30 sec, with a final extension step for 10 min at 72°C. The product was detected using electrophoresis and a 2% agarose gel (Sigma-Aldrich). The bands were scanned to analyze the gray values using a Smart Biological Electrophoretic Image Analyzer (Shanghai Furi Science & Technology Co. Ltd., Shanghai, China). The expression of MMP-2 was normalized to the corresponding GAPDH band (117 bp). The interference efficiency of MMP-2 was calculated according to the following formula: Interference efficiency = (1 - MMP-2 expression in the siRNA group/MMP-2 expression in the untreated group) × 100%.

### Analysis of siRNA-binding ability

The cationic liposomes were mixed with siRNA (0.01 g/l) at various mass ratios of cationic liposome to siRNA (0:1, 1:1, 3:1, 5:1, 7:1, 9:1 and 15:1) and incubated at RT for 30 min to allow for complete electrostatic interaction between the liposomes and siRNA. Subsequently, the complexes were obtained and loaded into an agarose gel to evaluate the siRNA-binding ability of the cationic liposomes. The bands were scanned to analyze the gray values using a Smart Biological Electrophoretic Image Analyzer. The binding abilities at each mass ratio were calculated according to the following formula: (Gray value at 0:1 - gray value at other mass ratio)/(gray value of 0:1) × 100%.

### Cell viability assay

An MTT assay was used to examine the cellular toxicity of the synthesized liposomes on HSC-T6 cells. The cells in the logarithmic growth phase were seeded on a 96-well plate (5×10^3^/well) and cultured for 24 h. Experimental wells were used for cationic liposome/siRNA complexes with different mass ratios, whereas wells containing fresh DMEM media and Lipofectamine 2000 were used as negative and positive controls, respectively. For each group, six parallel wells were prepared. Following incubation for 72 h at 37°C, MTT solution 20 *μ*l (5 g/l) was added to each well and after 4 h, the culture reaction was terminated using 150 *μ*l DMSO. The optical density values were read at 492 nm using a microplate reader (Thermo Multiskan MK3; Thermo Fisher, Vantaa, Finland), with the viability of the cells presented as the percentage compared with the negative control cells.

### Evaluation of transfection efficiency

The HSC-T6 cells were plated onto a 6-well plate (3×10^5^/well). At a density of ~50–60%, 0.2 ml cationic liposome/carboxyfluorescein (FAM)-siRNA complex with different mass ratios (0:1, 1:1, 3:1, 5:1, 7:1, 9:1, and 15:1, the concentration of siRNA was always 0.01 mg/ml) were added to the cells and then supplemented with 1.8 ml DMEM. Triplicate wells for the same condition were prepared. Lipofectamine 2000 and FAM-siRNA was used as a positive control, and FAM-siRNA alone was used as a negative control. After 6 h, the transfer efficiency was estimated using a fluorescent microscope.

### Gelatin zymography

The gelatin zymography procedure was described in detail in a previous study (12). Briefly, electrophoresis plates for 8% SDS-PAGE, including 1 g/l gelatin, were prepared (Sigma-Aldrich). The samples from the cultured supernatants were prepared using dialysis bags, with a molecular weight cut-off of 35 kDa, and incubated in polyethyleneglycol (Sigma-Aldrich) for 40 min at RT to concentrate the samples. Subsequently, 15 g concentrated supernatant from each sample was loaded for electrophoresis onto the prepared gel, which was run at 100 V. Subsequently, the gel was rinsed twice with Zymogram A buffer for 30 min at RT and incubated with Zymogram B buffer overnight. Finally, the gel was stained with Coomassie Brilliant Blue R-250 (0.2% Coomassie Brilliant Blue R-250, 20% methanol and 10% acetic acid) for 2 h at 37°C and destained in methanol to obtain clear bands. The gray values of the bands were analyzed using a Smart Biological Electrophoretic Image Analyzer. The MMP-2 activity was presented as a percentage of the activity of the 12 h control group.

### DAPI staining

The cells were fixed in cold acetone for 20 min, followed by staining with DAPI staining buffer for 10 min at RT. Following washing with PBS, the cellular morphology was analyzed and images were captured using a fluorescent microscope.

### Immunocytochemistry staining

The cells were fixed in cold acetone, and nonspecific antibody binding was blocked with goat serum. This was followed by incubation with the primary antibody, α-SMA, or type I collagen monoclonal antibody (1:100) and a secondary antibody, conjugated to biotin and immunoperoxidase with streptavidin at 4°C. Subsequently, the cells were visualized using DAB and H_2_O_2_, followed by counter-staining with hematoxylin (Sigma-Aldrich), and the intensity of staining was observed under a fluorescent microscope. The negative control was treated with PBS buffer instead of a monoclonal antibody. A total of five images were captured in the left, middle, right, upper and lower fields of each slide. The protein expression levels of α-SMA or type I collagen was calculated as the average percentage of positive cells (number of positive cells/number of total cells × 100%).

### Statistical analysis

All experiments were performed at least three times, and the numerical data are presented as the mean ± standard deviation. Statistical analysis was performed using SPSS 13.0 software. The difference between two groups were evaluated using Student's t-test for independent samples. P<0.05 was considered to indicate a statistically significant difference.

## Results

### Physicochemical properties of liposomes

**Figure 1:**
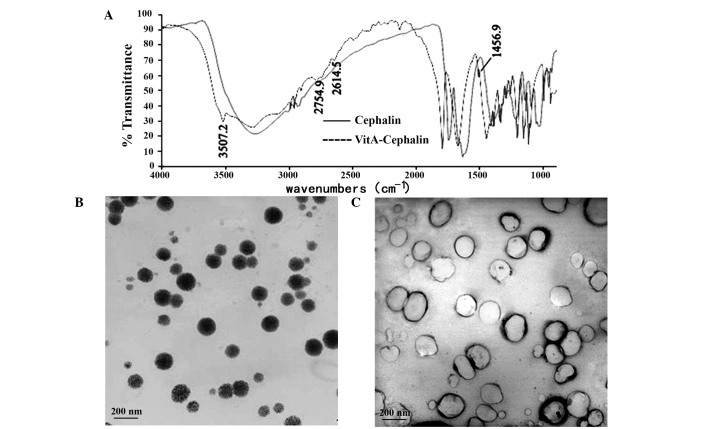
Characterization of cationic liposomes. (A) Stretching vibration peaks of cephalin modified with VitA on the Fourier infrared spectrum. Transmission electron microscopic images of (B) cationic liposomes and (C) VitA-Lips. VitA, vitamin A.

The present study used infrared spectra to examine whether cephalin was bound by VitA. The spectra of cephalin and VitA-cephalin were the same, with the exception of the vibration benzene peak at 1,456.9 cm^−1^ and an NH-stretching vibration characteristic peak of an amide bond at 3,507.2 cm^−1^ for VitA-cephalin, which confirmed that VitA bound cephalin via an amide bond (Fig. 1A).

Subsequently, the morphology and stability of the liposomes were examined. The results revealed that unmodified cationic liposomes appeared as white emulsions with light blue opalescence. They dispersed well, and the majority were single chamber and spherical-like, with particles sizes distributed between 100 nm and 200 nm (Fig. 1B). No flocculation or sediment were observed on being maintained at RT for 2 months, however, their diameter increased without prominent fusion. Following modification by VitA, the particle size of the cationic liposomes increased, and they exhibited mild aggregation (Fig. 1B). These findings were further confirmed by the results from the Zeta Sizer 3000 laser particle size analyzer. The data indicated that the average size of the cationic liposomes was 148.2±0.3 nm and the surface charge, based on the zeta potential measurement, was +41.67 mV, with these values changing to 227.3±4.1 nm and +44.67 mV, respectively following modification (Table I). The PDI values revealed an almost monodisperse particle size (Table I). These findings demonstrated that the VitA-lips exhibited a positive charges with good stability.

### Identification of MMP-2 siRNA interference efficiency

**Figure 2:**
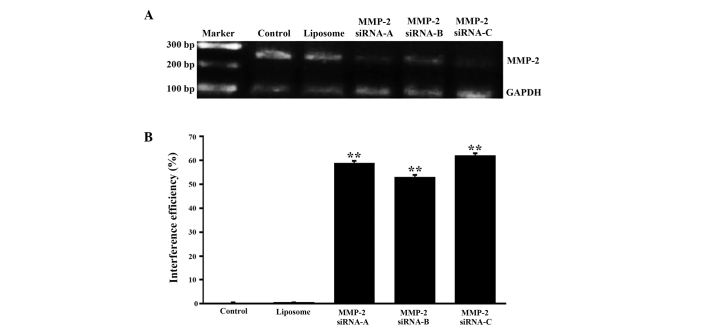
Interference efficiency of VitA-lip-MMP-2 siRNA complexes. The mRNA expression of MMP-2 was examined using reveres transcription-quantitative polymerase chain reaction in HSC-T6 cells. The cells were exposed to normal complete medium (control), liposomes and VitA-lip-MMP-2 siRNA complexes containing three designed MMP-2 siRNA-A/B/C sequences. (A) Images of gelatin zymography. (B) Quantitative analysis of densitometry indicating MMP-2 activity. The data indicated that the VitA-lip-MMP-2 siRNA complexes containing the MMP-2 siRNA-C sequence exhibited the highest interference efficiency. All the experiments were repeated at least three times. Representative images from one of the experiments are shown. Data are expressed as the mean ± standard deviation. ^**^P<0.01, compared with the untreated control. MMP-2, matrix metalloproteinase 2; siRNA, small interference RNA; VitA, vitamin A; lip, liposome.

To obtain higher gene silence efficiency, three interference sequences and corresponding random sequences of MMP-2 were designed. At 36 h post-transfection of the cells with the VitA-lip-MMP-2 siRNA complexes, the cells were used to evaluate the mRNA expression of MMP-2 using RT-qPCR. The data demonstrated that the interference efficiencies of MMP-2 siRNA-A, siRNA-B and siRNA-C were 58.76, 52.87 and 61.84%, respectively, and no interference effects were observed in the cells treated with VitA-lip only (Fig. 2). These results indicated that the mRNA expression levels of the target gene in the three interference groups were significantly different compared with that in the untreated control or VitA-lip group. Among these, MMP-2 siRNA-C exhibited the highest silencing efficiency and was used for the subsequent experiments.

### siRNA-binding capacity of theliposomes

**Figure 3:**
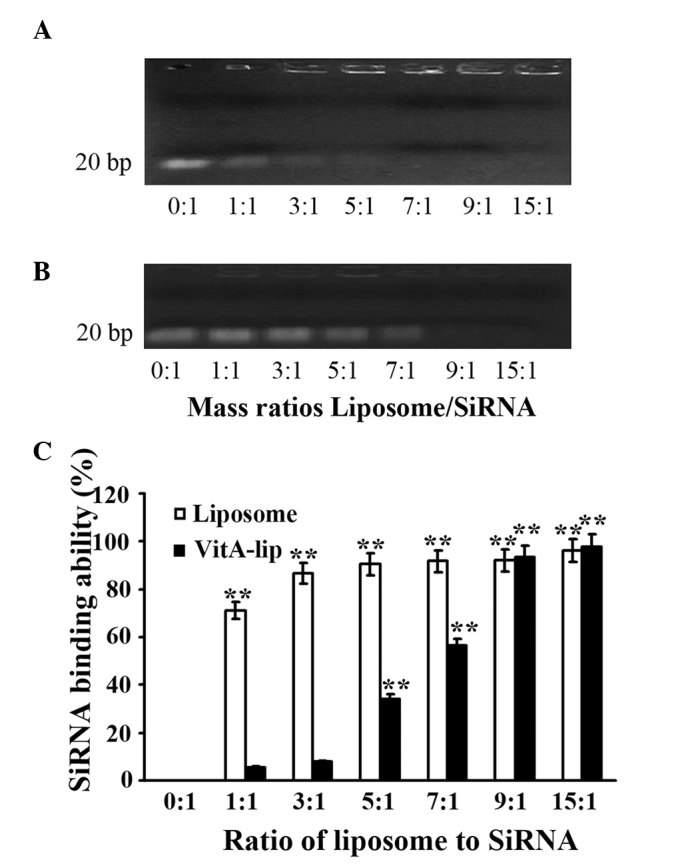
siRNA-binding capacity of cationic liposomes. Different mass ratios of cationic liposomes or VitA-Lips were mixed with siRNA, and a gel retardation assay was performed. (A) cationic liposomes; (B) VitA-Lips; (C) Quantitative analysis of densitometry in A and B. The mass ratios of liposome/siRNA were 0:1, 1:1, 3:1, 5:1, 7:1, 9:1 and 15:1 in lanes 1–7, respectively. The data are representative of three replicate experiments. Data are expressed as the mean ± standard deviation.^**^P<0.01, compared with the control (mass ratio, 0:1). siRNA, small interference RNA; VitA, vitamin A.

To determine the siRNA-binding capability of the liposomes, agarose gel electrophoresis was performed. This process demonstrated that the cationic liposomes were bound to more siRNA when the ratio of cationic liposome to siRNA was increased. The cationic liposomes completely encapsulated MMP-2 siRNA when the ratio of cationic liposomes to siRNA was ≥1. Following modifiication by VitA, the cationic liposomes effectively integrated with siRNA when the ratio was ≥5. Therefore, the cationic liposomes exhibited stable binding capability, which was efficient following modification by VitA (Fig. 3).

### Transfection efficiency of VitA-lip-MMP-2 siRNA

**Figure 4:**
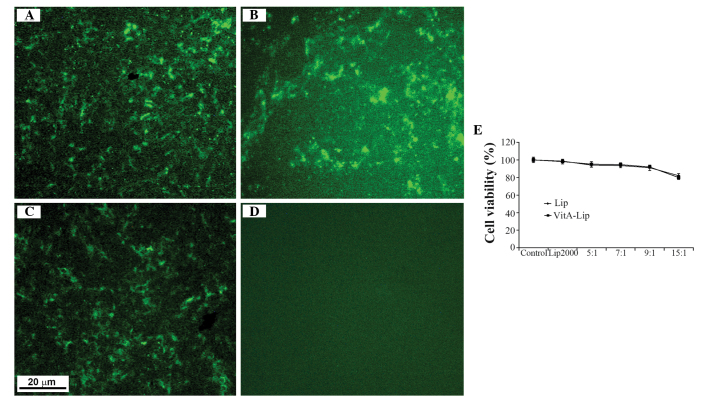
Transfection efficiency and cytotoxicity of VitA-lip-MMP-2 siRNA complexes. Cells were plated on six-well plates overnight and treated with (A) Lipofectamine 2000, (B) siRNA capsuled with cationic liposomes, (C) FAM-siRNA capsuled with VitA-lip and (D) FAM-MMP-2 siRNA After 6 h, the transfection efficiency was observed using a fluorescence microscope. (E) Cell viability was detected using an MTT assay following transfection of the cells with liposomes, or with MMP-2 siRNA encapsulated with unmodified cationic liposomes or VitA-lips. All experiments were repeated at least three times. Representative images from one of the experiments are shown. Data are expressed as the mean ± standard deviation. MMP-2, matrix metalloproteinase 2; siRNA, small interference RNA; VitA, vitamin A.

Prior to examining the transfection efficiency of the VitA-lip-MMP-2 siRNA complexes, their cellular toxicity was examined using an MTT assay. Following treatment of the cells with pure liposomes or with the complexes containing the various mass ratios of liposome to siRNA (5:1, 10:1 and 15:1), no cytotoxicity was observed in the HSC-T6 cells whether the complexes contained the liposomes or VitA-lips (Fig. 4E). As expected, the cytotoxicity was liposome concentration-dependent (Fig. 4E). Although decreased cell viability was observed in the HSC-T6 cells transfected with the complexes, they remained at ~80%, despite a liposome/siRNA ratio of 15:1 (Fig. 4E).

Subsequently, the cells were transfected with cationic liposome/MMP-2 siRNA complexes with different mass ratios and, after 6 h, green fluorescence was observed inside the cells exposed to the complexes, however no fluorescence was observed when cells were treated with MMP-2 siRNA alone (Fig. 4A–D). Among the cell groups, the highest transfection efficiency was detected when the ratio of liposome/siRNA was 5:1 prior to modification. Following modification with VitA, the most efficient transfection was achieved at a liposome/siRNA ratio of 7:1 (data not shown). These findings indicated that the transfection efficiency increased markedly following modification. However, the cellular toxicity was dependent on the mass ratio of liposome/siRNA, of which a ratio of 7:1 was selected for the subsequent experiments.

### Silencing efficiency of MMP-2 induced by VitA-lip-MMP-2 siRNA

**Figure 5:**
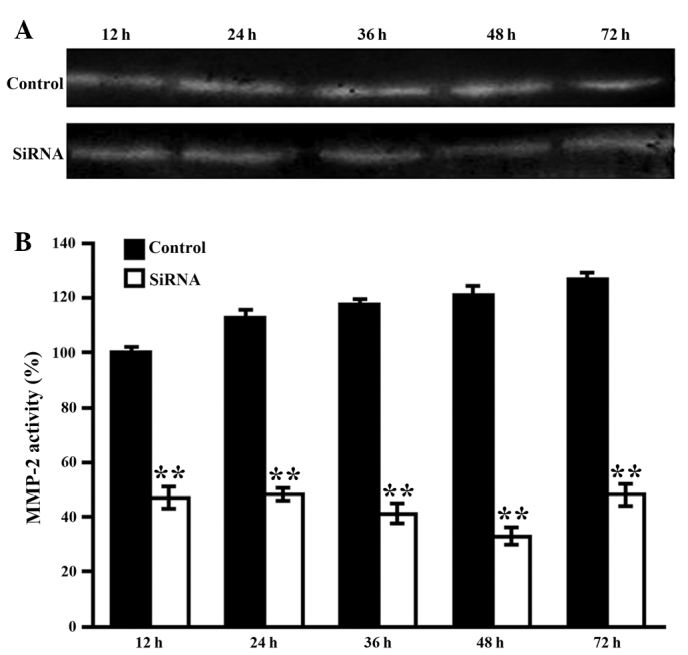
Interference efficiency of the VitA-lip-MMP-2 siRNA complexes. The MMP-2 activity of the cell culture supernatants was evaluated using zymography following transfection of the cells with VitA-lip-MMP-2 siRNA complexes for 12, 24, 36, 48 and 72 h. (A) Images of gelatin zymography. (B) Quantitative analysis of densitometry indicating MMP-2 activity. MMP-2 activity is presented as a percentage of the activity of the 12 h control group. All the experiments were repeated at least three times. Representative images from one of the experiments are shown. Data are expressed as the mean ± standard deviation. ^**^P<0.01, compared with the corresponding control. MMP-2, matrix metalloproteinase 2; siRNA, small interference RNA; VitA, vitamin A.

To further optimize the MMP-2 interference, the HSCs cells were transfected with VitA-lip-MMP-2 siRNA for 12, 24, 36, 48 and 72 h. The activities of MMP-2 in the supernatants were then detected using zymography. As shown in Fig. 4, the gene interference gradually increased between 12 and 48 h, peaked at 48 h and weakened at 72 h (Fig. 5).

### The effect of VitA-lip-MMP-2 siRNA on the cellular behavior of HSCs

**Figure 6:**
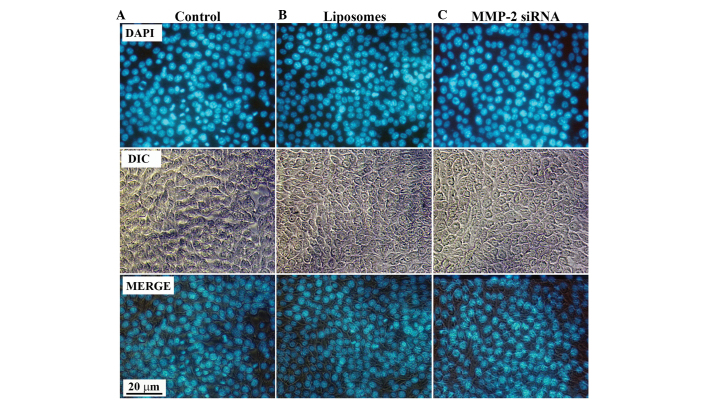
Effect of VitA-lip-MMP-2 siRNA complexes on apoptosis in the HSC-T6 cells. HSC-T6 cells stained using 4′,6-diamidino-2-phenylindole solution exhibited no clear apoptosis following transfection with MMP-2 siRNA for 48 h. (A) Standard culture medium; (B) liposomes; (C) VitA-lip MMP-2 siRNA. MMP-2, matrix metalloproteinase 2; siRNA, small interference RNA; VitA, vitamin A; DIC, differential interference contrast.

To investigate the effect of VitA-lip-MMP-2 siRNA complexes on the cellular behavior of HSC-T6 cells, DAPI staining was performed to examine whether apoptosis was induced. Following transfection of the HSCs with VitA-lip-MMP-2 siRNA complexes for 48 h, the cells were stained with DAPI staining solution. No differences in apoptosis were observed in the cells in the interference groups, compared with those of the control groups (Fig. 6), which suggested that the decreased viability of the HSC-T6 cells was due to the downregulated cell activation, and not apoptosis.

**Figure 7:**
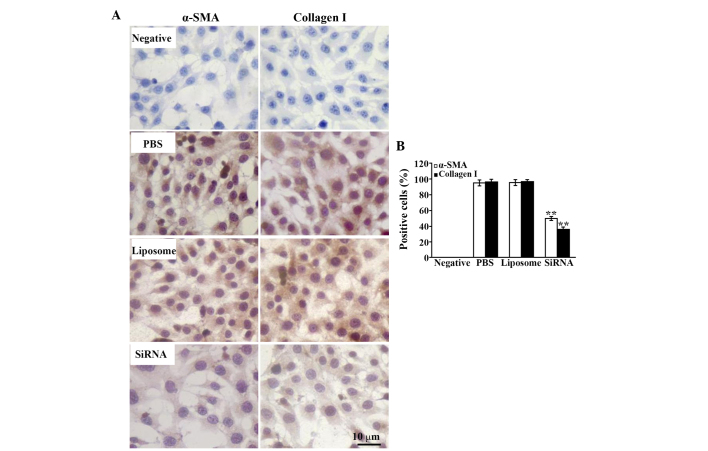
Effect of VitA-lip-MMP-2 siRNA complexes on HSC-T6 cell activation. The markers of activated HSCs, α-SMA and type I collagen, were evaluated using immunocytochemical staining in HSC-T6 cells treated with liposomes containing MMP-2 siRNA. (A) Representative fluorescent images. (B) Quantitative analysis of the changes in fluorescence intensity, indicating the degree of the activation in the HSC-T6 cells. Data are expressed as the mean ± standard deviation. ^**^P<0.01, compared with the untreated (negative group) or liposome group. HSCs, hepatic stellate cells; PBS, phosphate-buffered saline; VitA, vitamin A; siRNA, small interference RNA; SMA, smooth muscle actin.

To detect the activation of the HSC-T6 cells, the present study performed immunostaining with specific antibodies to α-SMA and type I collagen, with positive cells presenting diffusely distributed brown-yellow granules in the cytoplasm. The results demonstrated that the expression of α-SMA was significantly decreased and the number of positive cells was reduced following treatment with MMP-2 siRNA, compared with that of the control groups (Fig. 7). The same expression pattern was observed for type I collagen (Fig. 7).

## Discussion

Hepatic fibrosis is the final stage of all chronic hepatic diseases and may develop into cirrhosis and hepatic carcinoma, and finally induce liver failure (13). The sustained secretion of ECM is a prerequisite of hepatic fibrogenesis, and HSCs are vital cells, which produce ECM during the development of hepatic fibrosis (13,14). The activation of HSCs has been considered an important step for the formation and development of fibrosis (3,9,11,15). In the healthy liver, HSCs are in a quiescent state and are involved in the metabolism of VitA. Under physiological conditions, HSCs do not express α-SMA, and exhibit low proliferative ability and collagen secretion (10,11,16). However, when exposed to injury, HSCs are activated and transformed into a fibroblast phenotype (10,11). HSCs lose VitA in the cytoplasm, but express cytokines, receptors, α-SMA and ECM, including type I collagen, and proliferate rapidly (11,16,17). HSCs are not only key cells in ECM secretion, they also crucial cells in the generation of MMP (18). Previous reports suggest that hepatic fibrosis is reversible (1–3,6,14). However, no effective drugs have been developed against the formation of fibrosis in chronic liver disease due to the shortage of specific targets and drugs, and inevitable side effects.

MMP-2 is an important collagenase of the matrix metalloproteinase family and is produced by several types of cell, including activated HSCs (19). At the early stage of fibrosis, HSCs secrete substantial MMP-2 and degrade abundant type IV collagen around the HSCs, which promotes the further activation and proliferation of HSCs (11,12,16,20,21). The activated HSCs secrete more collagen and MMP-2, following which MMP-2 conversely accelerates HSC activation, which constitutes a malignant feedback loop. MMP-2 also contributes to angiogenesis, vascular remodeling and hepatic sinusoid capillarization, which aggravates the progression of fibrosis (20). However, in the naturally decaying stage, MMP-2 activity is increased (22). This indicates that MMP-2 promotes the development of fibrosis in the early stage, but induces the elimination of fibrosis in the late stage. These findings can assist in developing specific drugs for fibrosis by regulating the expression and activity of MMP-2 during different periods of hepatic fibrosis.

RNAi is a gene silencing tool of substantial functionality. To silence a specific gene, specific homogenous mRNA is degraded using double-stranded RNA. This technique has the advantage of high efficiency and specificity and is widely used for investigating prophylaxis and treatment of diseases (23). Considering the vital functions of activated HSCs in the development of hepatic fibrosis, several studies have examined the regulation of gene expression by siRNA interference, which further inhibits activation and proliferation, promotes apoptosis and increases degradation of the ECM in HSCs. The findings of these studies suggest that HSCs are promising targets in identifying effective drugs against hepatic fibrosis. However, the shortage of specificity in RNAi *in vivo* has limited its application. Thus, based on the specific receptor and regulating sequences on the cell surface, a number of vectors targeting HSCs have been identified, including mannose-targeted liposomes and folic acid-targeted liposomes (24). These vectors specifically deliver siRNA to HSCs, which protects healthy hepatocytes. Furthermore, the higher efficiency of siRNA transfer results in more effective therapy. Large numbers of VitA receptors are expressed on the cellular membrane of HSCs (11), and Sato *et al* confirmed its specificity and effectiveness *in vivo* (25). Therefore, the present study constructed VitA-coupled cationic liposomes to deliver MMP-2 siRNA, and the data confirmed that the vector efficiently conveyed siRNA into the HSCs and inhibited the gene expression of MMP-2. However, the specificity of the vector requires further investigation *in vivo*.

The HSC-T6 cell line, an activated HSC model, has been used as a target cell in several studies on hepatic fibrosis. HSC-T6 cells present the features of hepatic stellate cells and activation phenotypes, including vigorous proliferation, abundant expression levels of α-SMA and type I collagen, and fibroblast-like morphology (3,26). A study by Kawada (27) found that MMP-2 promotes the activation and proliferation of HSCs, activates the expression of α-SMA, a marker of HSC activation, and produces increased ECM, predominantly type I collagen. In the present study, HSCs activation and the expression of type I collagen were reduced following treatment with MMP-2 siRNA. The results demonstrated that the MMP-2 siRNA-transfected HSCs were smaller, and the ratio of nucleus to plasma was reduced. In addition, the cell viability was downregulated, which suggested that decreased activity and expression of MMP-2 reduced the the proliferation rate of the HSCs, however, these findings were not associated with apoptosis. Therefore, the present study hypothesized that the reduction in the viability of the HSCs may have been caused by MMP-2 siRNA-induced phenotype transversion of the activated HSCs. The immunostaining confirmed this hypothesis, as the protein expression levels of α-SMA and type I collagen were significantly decreased in MMP-2 siRNA-treated HSCs. However, whether the weakened viability of the HSCs is associated with the induction of senescence requires further investigation.

**Figure 8:**
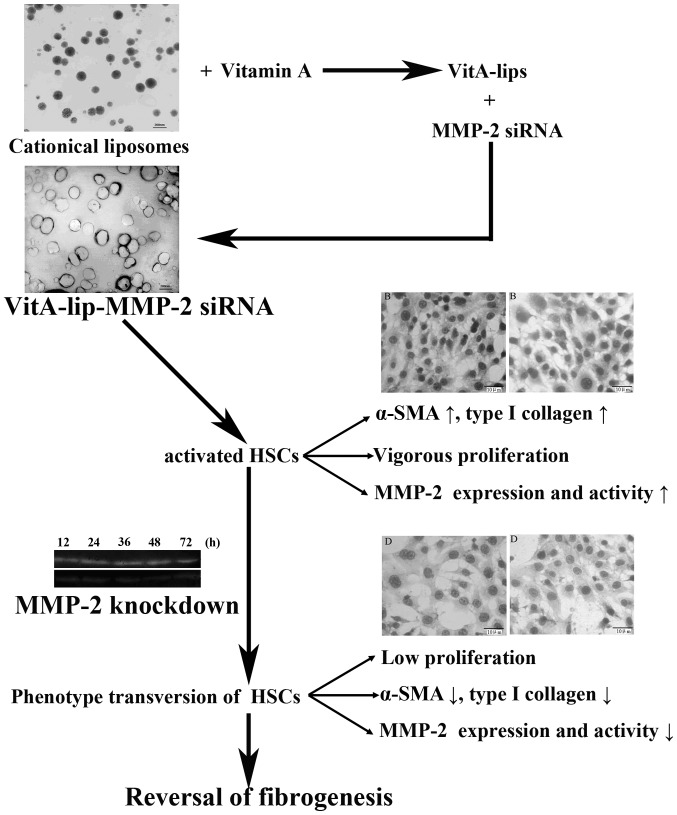
Schematic illustration of the association among HSCs, MMP-2 and fibrogenesis. At the fibrogenesis stage, hepatic stellate cells proliferate rapidly, secrete abundant MMP-2 and express markers of activated HSCs (α-SMA and type I collagen). However, when MMP-2 was downregulated by the constructed liposomes carrying MMP-2 siRNA, the viability of the HSCs decreased and expression levels of α-SMA and type I collagen were reduced. These results indicated that silencing MMP-2 reversed fibrogenesis. HSCs, hepatic stellate cells; PBS, phosphate-buffered saline; VitA, vitamin A; siRNA, small interference RNA; SMA, smooth muscle actin.

In conclusion, the present study successfully constructed a VitA-coupled cationic liposome vector, which effectively delivered MMP-2 siRNA to the HSCs. Following transfection with the vector, the expression and activity of MMP-2 in the HSCs were prominently downregulated. Based on these changes, the activation and proliferation of HSCs were decreased, and the secretion of type I collagen in the HSCs cells was significantly reduced (Fig. 8). These findings present a novel direction in the targeted prevention of hepatic fibrosis. However, further investigations are required to elucidate the application of this system *in vivo*.

## Figures and Tables

**Table I:** Characterization of liposomes.

Method	TEM	Zeta sizer 3000 laser particles analyzer		
	Size distribution (nm)	Z-average diameter (nm)	Polydispersity	Zeta potential (mV)
Liposome	114.34±36.94	148.2±0.3	0.162±0.014	+41.67
VitA-lips	155.77±39.00	2273±41	0.171±0.025	+44.67

Data are expressed as the mean ± standard deviation. VitA, vitamin A; lips, liposomes; TEM, transmission electron microscopy.
